# Improving Children’s Sleep Habits Using an Interactive Smartphone App: Community-Based Intervention Study

**DOI:** 10.2196/40836

**Published:** 2023-02-10

**Authors:** Arika Yoshizaki, Emi Murata, Tomoka Yamamoto, Takashi X Fujisawa, Ryuzo Hanaie, Ikuko Hirata, Sayuri Matsumoto, Ikuko Mohri, Masako Taniike

**Affiliations:** 1 Molecular Research Center for Children's Mental Development United Graduate School of Child Development Osaka University Suita, Osaka Japan; 2 Research Center for Child Mental Development University of Fukui Yoshida-gun, Fukui Japan; 3 United Graduate School of Child Development Osaka University Suita, Osaka Japan; 4 Department of Pediatrics Osaka University Hospital Suita, Osaka Japan; 5 Higashiosaka City Health Center Higashiosaka, Osaka Japan

**Keywords:** infant sleep, app, mHealth, mobile health, behavioral intervention, sleep health, social implementation, mobile phone

## Abstract

**Background:**

Sleep problems are quite common among young children and are often a challenge for parents and a hinderance to children’s development. Although behavioral therapy has proven effective in reducing sleep problems in children, a lack of access to professionals who can provide effective support is a major barrier for many caregivers. Therefore, pediatric sleep experts have begun developing apps and web-based services for caregivers. Despite the substantial influence of cultural and familial factors on children’s sleep, little effort has gone into developing cultural or family-tailored interventions.

**Objective:**

This study aimed to examine the effectiveness of the interactive smartphone app “Nenne Navi,” which provides culturally and family-tailored suggestions for improving sleep habits in young Japanese children through community-based long-term trials. The study also aimed to investigate the association between app-driven improvements in sleep and mental development in children.

**Methods:**

This study adopted a community-based approach to recruit individuals from the Higashi-Osaka city (Japan) who met ≥1 of the following eligibility criteria for sleep problems: sleeping after 10 PM, getting <9 hours of nighttime sleep, and experiencing frequent nighttime awakenings. A total of 87 Japanese caregivers with young children (mean 19.50, SD 0.70 months) were recruited and assigned to the app use group (intervention group) or the video-only group (control group). Both groups received educational video content regarding sleep health literacy. The caregivers in the intervention group used the app, which provides family-tailored suggestions, once per month for 1 year.

**Results:**

A total of 92% (33/36) of the caregivers in the app use group completed 1 year of the intervention. The participants’ overall evaluation of the app was positive. The wake-up time was advanced (base mean 8:06 AM; post mean 7:48 AM; *F*_1,65_=6.769; *P*=.01 and sleep onset latency was decreased (base mean 34.45 minutes; post mean 20.05 minutes; *F*_1,65_=23.219; *P*<.001) significantly in the app use group at the 13th month compared with the video-only group. Moreover, multiple regression analysis showed that decreased social jetlag (*β*=−0.302; *P*=.03) and increased sleep onset latency SD (*β*=.426; *P*=.02) in children predicted a significant enhancement in the development of social relationships with adults. At 6 months after the completion of the app use, all the caregivers reported continuation of the new lifestyle.

**Conclusions:**

The present findings suggest that the app “Nenne Navi” has high continuity in community use and can improve sleep habits in young Japanese children and that interventions for sleep habits of young children may lead to the enhancement of children’s social development. Future studies must focus on the effectiveness of the app in other regions with different regional characteristics and neuroscientific investigations on how changes in sleep impact brain development.

## Introduction

### Background

The Centers for Disease Control and Prevention, the National Public Health agency of the United States, described sleep deprivation as a “public health epidemic” linked to a wide range of medical issues, including hypertension, diabetes, depression, obesity, and cancer [1]. Sleep deprivation can impede not only physical health but also mental health and development.

Sleep problems are quite prevalent among young children, regardless of their cultural origins [2]. An international pediatric task force stated that insufficient sleep among children is a major public health concern [3]. According to a meta-analysis of sleep, cognitive, and behavioral problems in school-aged children (aged 5 to 12 years), inadequate sleep quality or quantity during childhood can affect daytime functioning, cognitive development, and health [4].

Current neuroscience research shows that the process of synaptic pruning occurs during rapid eye movement sleep [5], which demonstrates the importance of sleep from a developmental perspective. Recent cohort studies have shown that children who sleep less during infancy and early childhood are at a higher risk for hyperactivity and lower cognitive functioning later in their lives [6]. In addition, extant literature suggests that the early years (up to 3 years of age) are a sensitive period in which sleep can impact development [6,7]. A Norwegian cohort study investigating the link between sleep in early years and later development showed that short sleep duration and frequent nocturnal awakenings among toddlers aged 1.5 years were associated with the development of both internalizing and externalizing problems at 5 years of age [8]. Sivertsen et al [9] also found that short sleep duration (≤10 hours) and frequent (≥3) nightly awakenings at 1.5 years of age predicted the development of depressive symptoms at 8 years of age. A recent Australian cohort study also reported that sleep problems at 4 to 5 years of age are associated with internalizing difficulties through 12 to 13 years of age [10].

In addition, various studies have demonstrated that children with neurodevelopmental disorders are likely to have more sleep problems and that sleep parameters are associated with the severity of the symptoms of developmental disorders [11-13]. A recent article proposed a novel view on attention-deficit/hyperactivity disorder, whereby a part of the symptoms of attention-deficit/hyperactivity disorder were linked to chronic sleep disorders, with delayed circadian rhythm suggested as the underlying mechanism [14]. Furthermore, several studies on children with neurodevelopmental disorders have indicated that improvements in their sleep problems could lead to improvements in their behavioral problems [15-17]. However, in the context of the evidence regarding the association between sleep problems and developmental trajectories, there is a lack of clarity on whether sleep problems and the predictors of developmental disorders originally coexist or whether sleep problems in early childhood impact developmental trajectories [18].

Therefore, intervention studies examining early childhood are needed to determine whether the improvement of sleep in early childhood can prevent adverse outcomes and enhance healthy developmental trajectories [19]. We hypothesized that improving sleep problems in early childhood would result in better developmental trajectories, which is the ultimate goal of our research.

Furthermore, children’s sleep problems are associated with parental stress, family conflict, and maternal depressive symptoms [20-22] and are a risk factor for maltreatment [23]. The “Common risk assessment tool for child consultation centers and municipalities related to child abuse” issued by the Japanese Ministry of Health, Labour and Welfare in 2017 recommends that early support is needed for children with unstable sleep-wake rhythms and difficulty in sleeping [24]. The research also suggested that interventions for improving children’s sleep and developing good sleep habits in early childhood are likely to improve the quality of life for the whole family.

Previous studies support the substantial impact of cultural factors on children’s sleep habits [25-29]. In addition to cultural factors, caregivers’ lifestyles also impact children’s sleep habits [30]. Japanese infants and young children are reported to have the shortest sleep duration among the infants and young children among the 17 countries where the survey was conducted [31]. Our previous study revealed that 30.8% of preschool children are sleep deprived and that 56.7% of the caregivers of children who sleep <8 hours at night rated their children’s sleep as “good” [30]. It has been suggested that the current situation may be the result of Japan’s unique sleep culture, which values working hard over getting enough sleep; the working environment of caregivers; the living environment; and other complex factors along with a lack of sleep literacy. Furthermore, Japan has witnessed a rapid increase in the use of electronic devices and a shift to a more nighttime lifestyle in recent years, which threaten the sleep health and development of Japanese children. As infancy and early childhood are the sensitive periods for sleep, it is necessary for children to develop adequate sleep habits during their early childhood. However, given the various factors involved, improving the sleep health of Japanese toddlers can be quite challenging.

Recent findings suggest that parental factors both predict the outcomes of and are predicted by behavioral interventions for infant sleep problems [32]. Sviggum et al [33] suggested that early and customized guidance for caregivers, with a focus on revealing and acknowledging their experiences with sleep problems in their children, is essential in helping caregivers deal with the challenges [33]. Shetty et al [34] focused on daytime parenting and found that permissive or inconsistent daytime parenting practices were associated with more severe sleep problems [34].

Therefore, we can conclude that suggestions for caregivers should include guidance on daytime parenting practices and be tailored to the unique experiences of families by considering sociopsychological factors such as culture, values, family and housing environment, and the working situations of caregivers. This is particularly the case for Asia, where people have a habit of cosleeping; hence, it is necessary to provide culturally sensitive suggestions. Previous findings have suggested that there are various familial factors that can affect children’s sleep, such as sleep environment, wake-up time, delayed or irregular mealtime, screen time, physical activity, and irregular or late bedtime of parents [28,35-39].

In Japan, guidance on sleep and childcare has traditionally been provided through face-to-face consultations at public health care centers. However, as the number of dual-working families has increased, such consultations have become more difficult for caregivers, thereby limiting the availability of guidance. Recent studies have shown the efficacy of web-based and mobile health (mHealth) interventions for sleep problems in infants and young children [31,40,41]. However, these devices developed in Western countries cannot be used in Japan without substantial modifications to account for the cultural differences in sleep habits, such as cosleeping and sleeping on futon mattresses. Considering these background and previous reports, we developed a smartphone app called “Nenne Navi,” which facilitates interaction between caregivers and pediatric sleep experts to improve sleep habits in young Japanese children [42]. This app provides culturally and family-tailored advice to each family to make small behavioral changes in their lifestyles through the Plan-Do-Check-Act cycle (Multimedia Appendix 1). We previously conducted a community-based trial for 1 year. The aim of this study was to examine the app’s long-term continuity and effectiveness in improving children’s sleep habits and development and parental cognition and behavior.

### A Priori Hypotheses

The app demonstrates intervention adherence (continuity) for long-term use in the community-based trials and long-term effectiveness in improving the sleep habits of young children and the parenting efficacy of their caregivers.

## Methods

### Ethics Approval and Consent to Participate

This study was approved by the Osaka University Clinical Research Review Committee (CRB5180007) on January 23, 2017, before the start of the study. All the study procedures were conducted in accordance with the ethical standards of the Declaration of Helsinki. At the beginning of the baseline assessment, the participants received detailed information about the study’s goals and procedures and were informed about the underlying data protection. Written consent was obtained from all the participants individually. All participants received a coupon for books worth JAP ¥5000 (US $42) upon the completion of the trial. The amount was set to be increased to up to US $100 for the app group depending on their contribution. The participants in the app group were notified that they would receive additional rewards according to their app use but were not informed of the exact amount to avoid impacting the results.

### Participants

**Figure 1:**
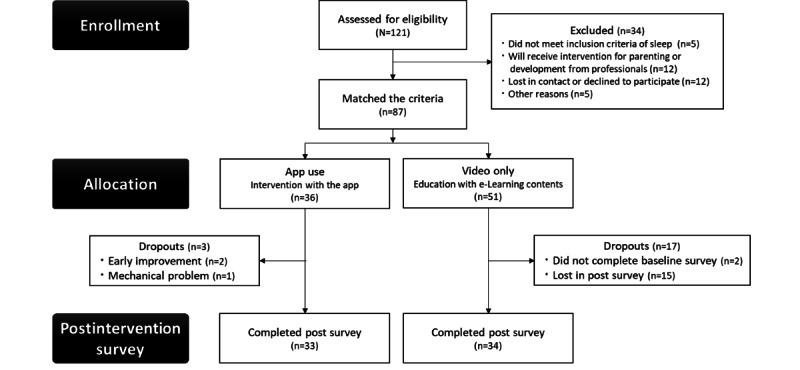
Flow diagram of the study.

A total of 87 Japanese caregivers (all mothers) with young children (mean 19.50, SD 0.70 months) from the Higashi-Osaka city were recruited over a 6-month period (September 2017 to March 2018) and assigned to either the app use group (intervention group) or the video-only group (control group) based on their preference; those without a preference were randomly assigned to either intervention. The Higashi-Osaka city is an urban area in the Western part of Japan with approximately 3000 births per year. It was confirmed that the sleep-wake patterns of the young children in this city were comparable with the national average in Japan (mean wake-up time: 7:12 AM, SD 0:58; mean bedtime: mean 9:20 PM, SD 0:54; Taniike et al, unpublished data, August 2016). Our study targeted caregivers with children who had completed the developmental health checkup at 1.5 years of age, as the app was designed for this age group. The inclusion criteria were that the children faced at least 1 of the following sleep problems: (1) bedtime later than 10 PM, (2) <9 hours of nighttime sleep, and (3) frequent night awakenings. In addition, the caregivers of the children needed to be fluent in Japanese, not currently be a participant of any interventions for parenting or children’s development, and be willing to participate in the study. The following inclusion criteria had to be met for the app use group: possession of a mobile device (iOS [Apple Inc] or Android [Google LLC]) with internet access and the willingness to install the “Nenne Navi” app on the mobile device. The following inclusion criteria had to be met for the video-only group: ability to access the internet to watch the video content and for record data. A total of 34 participants were excluded, as they did not meet the inclusion criteria. Supported devices included the iPhone, iPad, and iPod touch (Apple Inc) with iOS 8.0 or a later version, and Android devices with Android OS 4.3 or a later version. Both groups received educational video content regarding sleep health literacy. The caregivers in the app use group cooperated in the follow-up evaluation 6 months after the completion of the app use. A flow diagram of this study is shown in Figure 1.

### Measures

#### “Nenne Navi” App: Acceptability and Safety in Use

The Nenne Navi app was developed by pediatric sleep experts at the pediatric sleep clinic at Osaka University Hospital to positively influence caregivers’ behavior to ensure healthy sleep habits among their young children. The pilot trial of the app showed that there were no major problems with the system and that the usability and acceptability of the app was sufficient. Details of the system design were previously reported elsewhere by Yoshizaki et al [42]. The trademark was registered on July 20, 2018, and the registration number is 606435 “Nenne Navi” (Osaka University; MT, IM, Yoko Aoi, and AY). The patent number is 6920731.

The caregivers were asked to respond to approximately 36 questions regarding sleep-related lifestyles such as wake-up time, bedtime, nap time, screen time, daytime activity, dinner time, and bath time for 8 consecutive days via smartphone. Refer to Yoshizaki et al [42] for all the items in the app (Multimedia Appendix 2). The data were then sent to the Osaka University Virtual Server Hosting Service developed at Cybermedia Center, Osaka University, which is equipped with network security measures, such as access restrictions, encrypted communication, monitoring unauthorized access, and backup, to withstand cyberattacks. The system configuration was as follows: Ubuntu18+ apache 2.7, PHP 7, Postgre SQL9.

#### Individualized (Culturally and Family-Tailored) Intervention: Small Steps, Autonomous Choice of Behavioral Experiment, and Encouragement to Support Caregivers’ Motivation for Better Compliance

The pediatric sleep expert team consisted of 3 pediatricians and 2 psychologists, who analyzed the information entered by the caregivers and sent various types of practical advice to each caregiver. This app provides culturally sensitive advice on the parent-child lifestyle, such as wake-up time, bedtime, nap time, daytime activities, media use, dinner time, bath time, bedtime routine, and cosleeping habits, as problems with children’s sleep habits are caused by a variety of interrelated factors.

The app was designed with the ability to set individualized goals in accordance with individual users’ home lives; for example, the app sent personalized advice such as “Try to finish dinner before 8:00 p.m.” instead of “Try to have dinner earlier” to deliver specific and optimal goals to caregivers in small progressive steps based on the concept of behavioral therapy. The app does not expect caregivers to obey the advice; conversely, it was designed to send various pieces of advice and suggestions, from which caregivers could choose one to implement temporarily without much effort. Examples of the advice are presented in the study by Yoshizaki et al [42].

One of the features of the app allows caregivers to report the advice they have chosen and whether they have tried it on a monthly basis through the app. This enables the monitoring of caregivers’ spontaneous commitment to behavioral change, and the pediatric sleep experts can check the caregivers’ degree of compliance. A feedback message (of approximately 150 letters in English) was sent to each caregiver every month through the app to provide them with positive feedback on the improvements they have made in their lifestyle compared with the previous months. In addition, an icon in the app that displays an egg will gradually hatch and grow into a beautiful bird as users use the app. The app also has the function of plotting data into a graph. Caregivers can select their parameters of concern (eg, night awakenings and morning mood) in addition to the time their children fall asleep and see the changes visually. In addition to these personalized interventions and supports, the app provides all users with educational videos and tips on basic sleep and parenting literacy, specifically regarding positive daytime activities to create good sleep.

**Figure 2:**
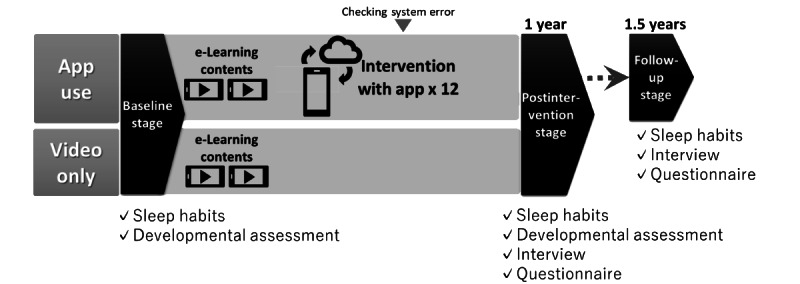
Intervention design of the study.

The design of the intervention study is shown in Figure 2.

#### Sleep Parameters

Data on sleep parameters was collected once a month for 8 consecutive days from baseline to the postintervention stage (1 year after) and at follow-up (1.5 years after) for the app use group, whereas for the video-only group, these data were collected for 8 consecutive days at baseline and 1 year after.

In addition to data on typical sleep parameters such as wake-up time, bedtime, nighttime sleep duration, and sleep onset latency, including their SDs, this study used data on social sleep restriction and social jetlag, as both these factors have been noted to be associated with physical health, mental health, and daytime performance. Social sleep restriction and social jetlag are defined as the difference in sleep duration between weekdays and weekends and shifts in sleep timing between weekdays and weekends, respectively.

#### Measure of Children’s Development

The parent-rated Kinder Infant Development Scale (KIDS) [43] was administered to all caregivers at the baseline and postintervention stages. The KIDS is a parent-rated questionnaire that was created in Japan to assess various aspects of the development of infants and toddlers, including motor, language, and social relationships.

#### Questionnaires and Interviews

A questionnaire was administered and semistructured interviews regarding the changes in parenting efficacy after app use were conducted following the trial to identify the effect of the app and the feasibility of its use in the community. The participants from both groups were asked to describe their impression of the educational video content and the changes in parenting efficacy. The participants in the app use group were additionally asked about their impression of the app, such as about the factors that motivated continued use. The participants responded to items asking them to evaluate the app and the video content using a 5-point scale (5=satisfied, 4=moderately satisfied, 3=neutral, 2=moderately dissatisfied, and 1=dissatisfied) and free descriptions. The participants responded to items asking them to evaluate the changes in parenting efficacy using another 5-point scale (5=very improved, 4=moderately improved, 3=unchanged, 2=moderately worsened, and 1=very worsened).

### Data Analysis

We conducted a 2-tailed *t* test to identify the between-group differences in sleep habits and sleep-related lifestyles of the participants in each group at baseline. We then conducted a 2-way ANOVA (group × time) to identify the efficacy of the app in improving children’s sleep habits (ie, bedtime, wake-up time, nighttime sleep, and sleep onset latency) via the community-based trial. A multiple regression analysis was conducted to evaluate the effects of improvement in sleep on young children’s development. Repeated measures analysis of variance was conducted to examine the maintenance of the effects of app use at follow-up. Data analysis was performed using SPSS statistics (version 26.0; IBM Corp).

## Results

### Demographic Information of the Study Participants

The demographic information of the study participants is presented in Multimedia Appendix 2. There were no between-group differences, except in fathers’ educational status.

### Improvement in Sleep-Wake Patterns in Children

The sleep habits and sleep-related lifestyles of the participants at baseline are provided in Table 1.

The app use group displayed significantly later wake-up times, longer sleep onset latency, larger SD for wake-up time, bedtime, and nighttime sleep duration, larger SD for sleep onset latency, and larger social jetlag compared with the video-only group at baseline. Furthermore, the app use group displayed a tendency to have more bedroom feeding habits and delayed wake-sleep rhythm. It is possible that a bias existed in which the caregivers of children with poorer sleep habits preferred the app use group.

**Table 1:** Sleep habits and sleep-related lifestyles of the participants in each group at baseline^a^.

	App use group, mean (SD)	Video-only group, mean (SD)	*P* value
Wake-up time (time, minute)	8:06 AM (0:55)	7:20 AM (0.48)	*<.001^b^*
Wake-up time SD (minutes)	38.95 (18.51)	30.26 (21.33)	.08
Bedtime (time, minute)	9:36 PM (0:59)	9:14 PM (0:49)	.097
Bedtime SD (minutes)	36.02 (23.71)	23.35 (13.16)	*.01*
Sleep onset latency (minutes)	34.45 (21.45)	22.01 (18.45)	*.01*
Sleep onset latency SD (minutes)	20.72 (11.70)	11.86 (11.00)	*.002*
Nighttime sleep duration (minutes)	593.52 (46.55)	583.59 (38.94)	.35
Nighttime sleep duration SD (minutes)	53.93 (25.16)	39.23 (21.44)	*.01*
Number of awakenings after sleep onset	0.99 (1.03)	0.76 (1.13)	.40
Nap starting time (time, minute)	1:41 PM (1:17)	1:22 PM (0:50)	.23
Nap starting time SD (minutes)	97.20 (54.71)	80.47 (37.92)	.15
Nap ending time (time, minute)	3:53 PM (1:20)	3:26 PM (0:57)	.12
Nap ending time SD (minutes)	86.35 (37.70)	78.09 (38.81)	.38
Nap duration (minutes)	106.52 (35.98)	104.79 (25.07)	.82
Nap duration SD (minutes)	39.08 (21.37)	38.77 (17.19)	.95
Total sleep duration (minutes)	700.00 (50.50)	688.35 (42.64)	.31
Total sleep duration SD (minutes)	59.15 (24.78)	48.81 (24.84)	.09
Television-viewing time (minutes)	118.70 (100.35)	87.47 (69.98)	.15
End of television-viewing time after 4 PM (time, minute)	8:37 PM (1:45)	8:31 PM (1:17)	.79
Smartphone-use time (minutes)	11.82 (18.00)	7.62 (19.06)	.36
End of smartphone-use time (time, minute)	5:35 PM (3:13)	5:44 PM (2:33)	.89
Outdoor play in the morning (%)	57.42 (37.65)	68.00 (37.93)	.26
End of dinner (time, minute)	7:22 PM (0:48)	7:15 PM (0:43)	.52
End of bathing (time, minute)	7:54 PM (1:27)	7:34 PM (1:41)	.38
Breastfeeding in the bed (%)	32.48 (45.95)	11.35 (28.76)	*.03*
Media use in the bed (%)	2.15 (6.32)	0.85 (4.97)	.35
Caregiver wake-up time (time, minute)	7:39 AM (0:53)	6:39 AM (0:48)	*<.001*
Caregiver bedtime (time, minute)	11:30 PM (1:20)	10:52 PM (1:20)	.06
Caregiver sleep onset latency (minutes)	39.59 (26.18)	31.31 (30.91)	.39
Caregiver nighttime sleep duration (minutes)	449.88 (54.11)	435.62 (77.23)	.24

^a^Differences between groups were tested using the 2-tailed *t* test.

^b^Italicized values indicate significance.

**Figure 3:**
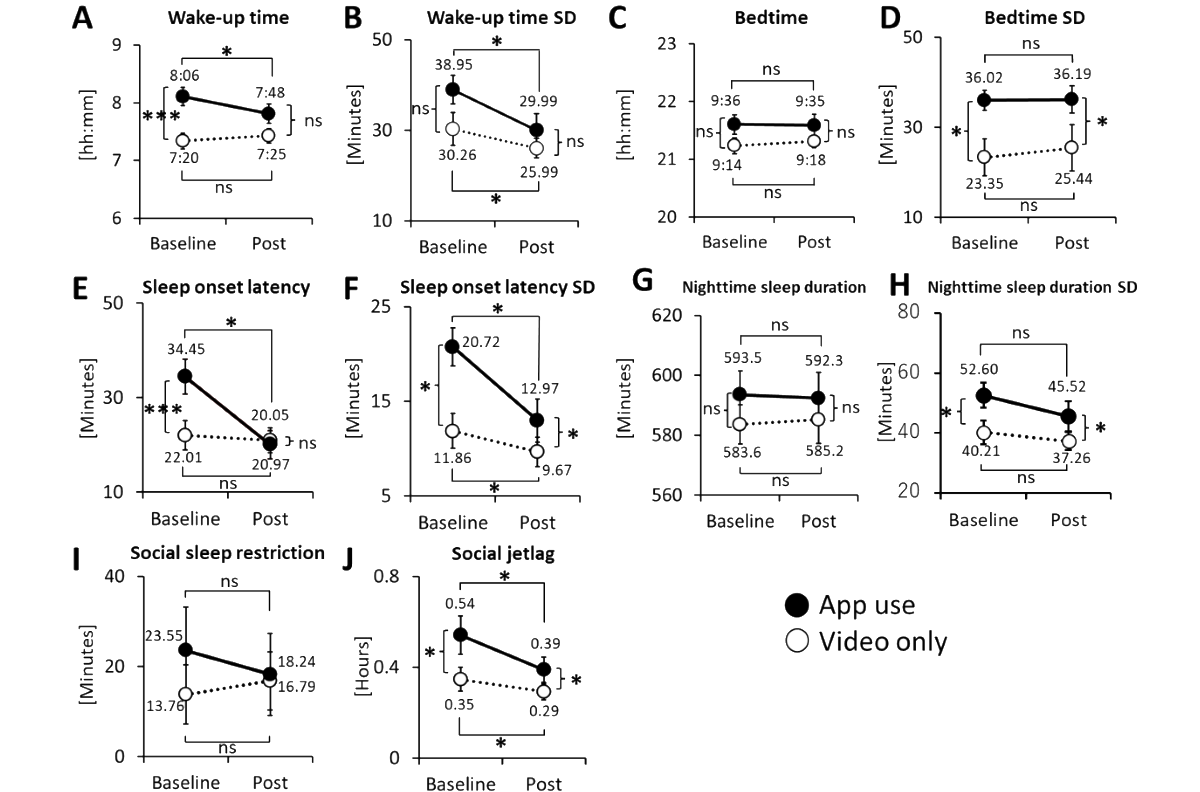
Children’s sleep-wake patterns by group.

A summary of the results is presented in Figure 3. Two-way ANOVAs were conducted to determine the effects of the group (the app use group and the video-only group) and intervention (baseline and post) on the sleep habit variables. First, significant interactions between the effects of the group and intervention were confirmed for wake-up time (Figure 3A; *F*_1,65_=5.748; *P*=.02) and sleep onset latency (Figure 3E; *F*_1,65_=12.389; *P*<.001), with only the app use group showing reductions for both outcomes (*P*=.05). Next, significant main effects of both the group and intervention were confirmed for sleep onset latency SD (Figure 3F; group: *F*_1,65_=8.037, *P*=.006; intervention: *F*_1,65_=7.969, *P*=.006) and social jetlag (Figure 3J; group: *F*_1,65_=4.264, *P*=.04; intervention: *F*_1,65_=4.709, *P*=.03). Furthermore, only the main effect of the intervention was observed for wake-up time SD (Figure 3B; *F*_1,65_=6.413; *P*=.01), whereas only the main effect of the group was observed for bedtime SD (Figure 3D; group: *F*_1,65_=6.991; *P*=.01) and nighttime sleep duration SD (Figure 3H; group: *F*_1,65_=5.510; *P*=.02). Finally, there were no significant main effects or interactions for bedtime (Figure 3C; *F*_1,65_=2.429; *P*=.12), nighttime sleep duration (Figure 3G; group: *F*_1,65_=.743; *P*=.39), or social sleep restriction (Figure 3I; *F*_1,65_<0.449; *P*=.51).

### Feasibility of Using Nenne Navi in the Community

There were no dropouts at the 6-month point, and only 8% (3/36) of the caregivers in the app use group dropped out after 1 year of the intervention. A total of 6% (2/36) of caregivers decided to finish the intervention prematurely, as they determined that they had “improved enough” at the 6-month point, based on the disappearance of night awakening (mean: more than 5 times per night before the intervention to none after), reduction of sleep onset latency as the changes for each of the 2 children who finished intervention prematurely (mean 27.9 minutes to “immediately”), reduction of sleep onset latency (mean: from 20 minutes to 5 minutes), and bedtime refusal. Overall, 3% (1/36) of caregivers dropped out of the intervention owing to internet connectivity issues on her smartphone. The mean score for the evaluation of the app was 4.303 (SD 0.969) and that for the evaluation of the video content was 4.545 (SD 0.782). In the feedback section on the app, the participants mentioned the following as the factors that encouraged continued app use (multiple answers allowed): (1) 91% (30/33) of the caregivers mentioned “caring and encouraging text messages to caregivers,” (2) 64% (21/33) mentioned “individually tailored advice,” (3) 18% (6/33) mentioned “growth of the bird icon,” and (4) 9% (3/33) mentioned “nothing in particular,” and (5) 3% (1/33) mentioned “other reasons.” Regarding their perception of the appropriate duration of app use, 42% (14/33) of the caregivers answered that a period of around 6 months would be appropriate, 52% (17/33) answered “10–12 months,” and 6% (2/33) answered “13 months or longer.” At the postintervention stage, of the 33 caregivers, 1 (3%) caregiver stated, “At first, I thought it would be impossible to achieve the goal of turning off the TV at 7:00 PM. But as I worked on other goals and adjusted my lifestyle, I gradually started to turn off the TV earlier and earlier, and finally I made it!!”

### Improvement in Parenting Efficacy

In terms of the improvement in the parenting efficacy of the participants, the mean score was 3.8 (SD 1.1) in the app use group and 3.3 (SD 1.0) in the video-only group. The results of the independent *t* test comparing the 2 groups were as follows: *t*_62_=1.996, *P*=.05. In the app use group, 32% (10/31) of the caregivers rated their parenting efficacy after intervention as “very improved” and 32% (10/31) as “moderately improved,” whereas in the video-only group, 6% (2/33) of the caregivers rated their parenting efficacy after intervention as “very improved” and 46% (15/33) as “moderately improved.” A chi-square test was performed to compare the proportion of caregivers in both groups who responded for the score of change in parenting efficacy. The results showed a trend for differences in response between the groups (*χ*^2^_4_=8.3; *P*=.08; *φ*=0.361). A residual analysis revealed that the caregivers of the app use group selected the “very improved” option significantly more than the video-only group (*P*=.01).

### Association Between the Content of and Preference for Advice

**Figure 4:**
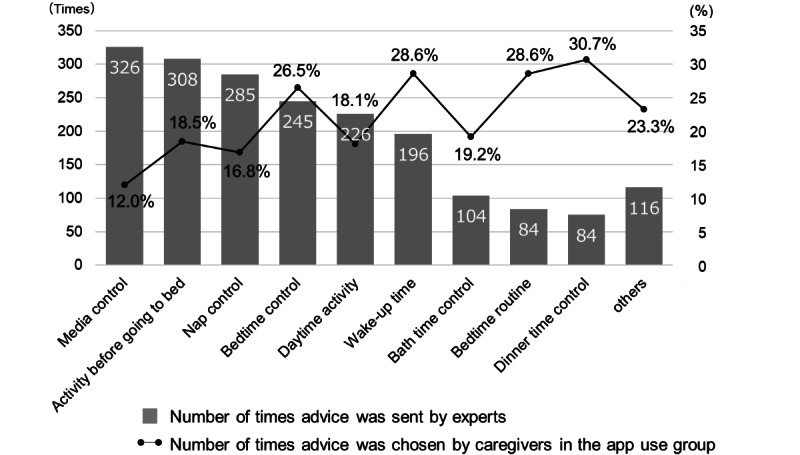
The advice was sent by the experts and chosen by the caregivers to try.

Figure 4 shows data on the advice sent by the pediatric sleep expert team to the caregivers and the advice chosen by the caregivers. The app offers a variety of advice concerning the daily lives of children and parenting behavior. There was a discrepancy between the advice that the experts tended to focus on (send frequently) and the advice that the caregivers tended to choose to try. The most commonly sent advice was about media control at home; however, it was the advice that the caregivers chose the least. The caregivers were most likely to select the advice about controlling dinner time. The “Others” category included advice on household chores (eg, chores around bedtime should be done after the children have slept or next morning), iron intake for children who are suspected of having restless leg syndrome, receiving help from family members, and adopting measures to maintain a healthy lifestyle. When the advice was sent, the following cultural issues were taken into account: bed sharing with caregivers and siblings, breastfeeding while lying in bed as a bedtime routine, awaiting father’s return home for dinner or bathing, and breastfeeding at midawakening while lying in bed.

### Effects of Changes in Sleep Habits on Mental Development

We performed a multiple regression analysis to examine the relationship between the changes in sleep habits and mental development to clarify the effects of the changes in sleep habits from baseline to postintervention stage on children’s development. Developmental age scores in each group, as assessed using KIDS, at baseline and postintervention stage at baseline and postintervention stage are shown in Multimedia Appendix 3. Table 2 shows the results of the multiple regression analysis. The changes (Δ) in sleep parameters from baseline to the postintervention stage were entered as predictors. Multiple regression analysis showed that the model was significant for social relationships with adults (*F*_1,64_=2.399; *P*=.02; *R*^2^=0.303) but not for social relationships with children (*F*_1,64_=1.118; *P*=.37; *R*^2^=0.169). Increased sleep onset latency SD and decreased social jetlag were significant predictors of the development of social relationships with adults (*β*=.426, *t*_54_=2.521, *P*=.02; *β*=−0.302, *t*=−2.883, *P*=.03).

**Table 2:** Multiple regression analysis of social development with sleep habit variables as predictors.

Independent variables	B (SE; 95% CI)	*β*	*t* test (*df*)	*P* value
Wake-up time (Δ)	−2.092 (4.011; −10.130 to 5.946)	−0.281	−0.522 (54)	.60
Wake-up time SD (Δ)	0.025 (0.037; −0.048 to 0.098)	.107	0.681 (54)	.50
Bedtime (Δ)	3.606 (4.085; −4.581 to 11.794)	.434	0.883 (54)	.38
Bedtime SD (Δ)	−0.033 (0.035; −0.104 to 0.038)	−0.165	−0.941 (54)	.35
Sleep onset latency (Δ)	0.043 (0.072; −0.101 to 0.188)	.160	0.601 (54)	.55
Sleep onset latency SD (Δ)	*0.145 (0.039; 0.068 to 0.223)* ^a^	*.426*	*2.521* *(54)*	*.02*
Nighttime sleep duration (Δ)	0.042 (0.061; −0.081 to 0.165)	.354	0.686 (54)	.50
Nighttime sleep duration SD (Δ)	0.022 (0.035; −0.048 to 0.091)	.119	0.633 (54)	.53
Social sleep restriction (Δ)	−0.019 (0.010; −0.040 to 0.001)	−0.250	−1.912 (54)	.06
Social jetlag (Δ)	−*4.166 (1.445; −7.053 to 1.278)*	−*0.302*	−*2.883 (54)*	*.03*

^a^Italicized values indicate significance.

### Long-term Effects of the App Intervention

A total of 28 (78% of caregivers in the baseline population) caregivers from the app use group participated in the follow-up, including the individual interview months after the end of the intervention. We conducted a repeated measures ANOVA of each sleep parameter at baseline, postintervention, and follow-up time points to examine the follow-up effects of app use. No correction was performed for the missing values. Multimedia Appendix 4 shows the sleep-wake patterns of the children in the app use group at these 3 time points. Wake-up time progressively and significantly advanced from baseline to follow-up. Wake-up time SD, sleep onset latency, sleep onset latency SD, and social jet lag were also significantly shortened between baseline and follow-up. There were no sleep parameters that showed a significant deterioration at follow-up.

As for the caregivers’ subjective responses in the follow-up, about 80% (22/28) of the users who participated in the follow-up answered that “the effect of improving sleep habits is still continuing,” implying a maintenance of the effect of the app. Furthermore, about 30% (8/28) of app users who participated in the follow-up answered that they began to offer advice to other caregivers around them who were facing problems with their children’s sleep.

## Discussion

### Principal Findings

This study investigated the effectiveness of the interactive smartphone app “Nenne Navi,” which provides culturally tailored and family-tailored suggestions for improving the sleep habits of young Japanese children. The results of a long-term trial with community populations suggested that “Nenne Navi” has a very high continuity and that the use of the app can contribute to the promotion of changes in parenting behavior, healthy sleep habits, and development in children. It is also suggested that the improvement in sleep habits resulting from the use of this app might be maintained after the end of the intervention, although further examination of the long-term effects is needed. One of the goals of remote interventions such as mHealth or computerized cognitive behavioral therapy is to minimize dropouts and maintain intervention adherence—high dropout rates, which can reach up to 80%, have been one of the main issues plaguing remote cognitive behavioral therapy [44-47]. Longer programs were associated with higher dropout rates in previous research [48]. The continuity of Nenne Navi was remarkably high in the community-based trial, as there was no dropout at the 6-month mark, and only 8% (3/36) of the participants in the app use group dropped out after 1 year. This high compliance may be explained by the design of the app, which endeavored to promote the confidence and empowerment of caregivers. Some of the design aspects to which the high intervention compliance can be attributed are (1) a reciprocal intervention design instead of a 1-way instruction based on valid suggestions and information; (2) thorough caregiver support function throughout the intervention, such as encouraging and motivating text messages for caregivers; (3) adequate sleep literacy via educational video content; (4) an intervention system that can increase the caregivers’ proactive commitment by sending advice several times corresponding to each individual situation and letting the user choose 1 item “to try”; (5) providing small-step multiple advice that can help bridge the gap between “proper literacy” and “caregivers’ limitations” while supporting parenting efficacy; and (6) the use of unique design features, such as the growth of the egg icon in response to use and a graphical representation of the parameters selected by individual caregivers. These elements conform to the suggestions of Whittall et al [49]. Surprisingly, as we confirmed the factors that motivated the continued use of the app, many caregivers reported that “caring and encouraging text messages” were the driving force behind their continued use.

Significant between-group differences in sleep-wake patterns were observed at baseline. Overall, the app use group displayed worse sleep habits at baseline. These differences may be explained by a tendency among caregivers more troubled by their children’s sleep problems to prefer being recruited to the app use group. Surveys in Japan have shown that wake-up time does not advance in the natural developmental process without intervention among children in the age range covered in this study [50,51]. Regarding the significant within-group change from baseline to the postintervention stage, the improvement in wake-up time and sleep onset latency in the app use group from baseline to the postintervention stage was owing to individual intervention and not the natural course.

The improvement in wake-up time SD, sleep onset latency SD, and social jetlag in both groups may have been the result of the education on “constant sleep rhythm” provided by the educational video delivered to both groups, which led to an improvement in rhythm irregularity.

Overall, this app was effective in improving wake-up rhythm and reducing the difficulty in falling asleep. The improved wake-up rhythm could be explained by the fact that it is relatively easy for mothers to wake up their children at a certain time, as they do not need the cooperation of other family members and already have practice in doing this. Intervention on wake-up time would be important because delayed wake-up time leads to delayed timing of nighttime melatonin secretion as well as napping rhythms, which will inevitably lead to reduced sleep pressure and delayed bedtime at night. In addition, improvement in waking time is also considered beneficial for adaptation to social life. Furthermore, the shortened sleep onset latency was attributed to the increased sleep pressure caused by the advancement of the wake-up rhythm, as well as the guidance provided by the app on establishing bedtime routines and sedative ways of spending time before going to bed, which are relatively new concepts in Japan. Nonetheless, effects on bedtime and extension of sleep time were not observed in this study. It might have been difficult to advance bedtime, as many factors are required to accomplish this. For example, several caregivers reported that it was difficult to obtain the cooperation of other family members in controlling the media use to advance bedtime or bath time. In addition, because many children in Japan go to bed together with their caregivers, the bedtime tended to be late, as the caregivers (the mothers in most cases) had to finish all the household chores before sleeping. However, it must be noted that the trial was conducted in a single community in a relatively urban area and, therefore, may reflect living conditions specific to the region, such as a greater exposure to light at night.

Consequently, although the children in the app use group made significant advances in wake-up time without advances in bedtime, their nighttime sleep duration was not shortened because of decreased sleep onset latency. As sleep duration did not change, there may have been no change in social sleep restriction. The development of further strategies to improve bedtime and sleep duration is an important focus area for future intervention studies.

Allen et al [52] conceptualized the elements of adequate or good sleep for children in a review of studies examining sleep regularity, bedtime routines, quiet or noise comfort or lights, media use, activities, and family conflict. They identified that there are many factors that impact children’s sleep. Recent findings suggest that there are multiple barriers for caregivers to adjust parenting behavior to create better sleep habits or reduce sleep problems [49]. The regional characteristics suggest that Japanese caregivers face relatively more barriers to accessing professional help when their children face trouble sleeping, as there are only a few pediatric sleep specialists in Japan. Thus, Japanese caregivers have little access to sleep literacy or solutions to their children’s problems. The dissemination of “children’s sleep literacy” is an urgent and critical social issue. In addition, the need for remote intervention tools has been further heightened by the COVID-19 pandemic. The COVID-19 pandemic has created new barriers for families struggling to raise their young children and might leave many families isolated. Thus, a means for pediatric sleep specialists to safely communicate up-to-date knowledge without face-to-face contact to caregivers can aid in bridging these barriers and bring many benefits to families.

This app helps caregivers to overcome some of the possible barriers through its unique design incorporating integrated support and small-step care intervention strategies. It is necessary for busy caregivers and those who lack adequate help or knowledge regarding childcare to achieve appropriate levels of sleep literacy and receive positive feedback to improve their children’s sleep habits. Many caregivers will benefit from this design aimed at empowering them to change their parenting behavior. Bradway et al [53] pointed out that it is essential to focus on the impact on users’ self-efficacy and engagement when judging the success, usefulness, and potential benefits of mHealth in health intervention research [53]. Our findings also suggest that designing the app to increase user engagement and self-efficacy contributed to high continuity of use and effectiveness.

Interestingly, there was a discrepancy between the advice that the experts selected on the basis of scientific priority and the advice that the caregivers chose to try. The greater discrepancy in the media control goal might accurately reflect the reality of parenting. Sleep and media use in young children have been a concern for many sleep professionals overseas [54,55]. However, in recent years, children have been reported to be exposed to media devices at increasingly younger ages, and the guidelines on screen time published by the American Academy of Pediatrics [56] appear to be ignored by approximately 90% of caregivers [57]. Some studies suggest that excessive media use may negatively impact brain development [57-59]. Media control at home is a major concern in Japan, similar to other high-income countries, and is often not practiced despite caregivers’ knowledge of the potential harm caused by excessive media use. Social transitions, such as an increasing number of nuclear families and dual-earner families, necessitate caregivers to rely on visual media to keep their children busy so that they can focus on household chores. Nonetheless, interviews with caregivers revealed that one of the most effective pieces of advice for improving sleep habits was media control (data not shown). Although the issue may be difficult to solve, this study clarifies that it is an area that professionals should focus on to help “build the bridge” to improve children’s sleep. As this app emphasizes the empowerment of caregivers and their own spontaneous behavioral changes, we designed it to build up “what I can do now” scenarios one by one. Regarding advice that is considered important by experts for improvement but not chosen by caregivers, we may be able to help them by providing practical tips or other support for carrying it out; additionally, we should be considerate of the possibility that there may be some limitations (eg, housing conditions) depending on familial situations.

In accordance with the previous reports that sleep in childhood was associated with later socioemotional problems [8,60], we focused on the social relationships with children and adults as a socioemotional developmental index. We found that decreased social jetlag and increased sleep onset latency SD in children predicted significant enhancement of social development. Although there are many indications of the adverse health effects of social jetlag in adolescents and adults [61,62], the results of this study suggest that close attention should be paid to social jetlag in young children from a developmental perspective. A recent study reported that social jetlag is negatively associated with serum brain-derived neurotrophic factor levels, which play an important role in neuronal maintenance, plasticity, and neurogenesis [63]. The age of the participants in this study, from 1.5 years to 2.5 years, is regarded as an age of remarkable socioemotional development (critical or sensitive period) in the trajectories of brain development [64,65]. The age of 2 years has been defined as a time when the restructuring of the parent-child relationship progresses from the perspective of a developmental theory of parent-child attachment [66]. Our results suggested the possibility that a reduction in social jetlag during this period might play a role in the enhancement of social development. Further research is needed to explain why the social jetlag could be related to social development in young children. Our results also suggested that the increased sleep onset latency SD might be also related to social development with adults. One possible explanation for this association is that as caregivers begin to modify their living situations and bedtime routines to accommodate better sleep, sleep onset latency might range from being very short (successful days) to long (unsuccessful days for any reason), which could have contributed to the current results. This association has not yet been clarified in many aspects and needs to be further investigated in the future.

In Japan, children’s sleep habits are strongly influenced by the sleep habits of their caregivers owing to the cosleeping lifestyle. Fukumizu et al [67] suggested that the cosleeping habit and bedtime irregularity were associated with sleep-related nighttime crying in Japanese children. It is necessary to increase awareness among families and help parents make changes in parenting to ensure that their children are not negatively impacted by irregular sleep habits. The relationship between increased sleep onset latency SD and a promotion of social development with adults remains to be clarified; however, there are some possible explanations. First, it may have been related to caregivers’ efforts to change parenting behaviors to help children sleep. For example, some caregivers discontinued breastfeeding as a bedtime routine and started reading picture books instead. Although the range of sleep onset latency increased and then varied temporarily, it may have had a positive impact on the parent-child relationship. Alternatively, children who were sleep deprived and fell asleep immediately at a later bedtime at baseline went to bed earlier with varied sleep onset latency.

The use of an interactive design in the app demonstrated its similarity to precision medicine, which can identify the issues in each family and provide optimized suggestions. Recent findings suggested that there are multiple barriers or reasons for caregivers not seeking help for children’s sleep problems, indicating that deferential help-seeking interventions are needed depending on the barriers or problem severity [68]. Increasing the choice of caregiver education and interventions, such as video-based caregiver education, app-based individualized remote interventions adopted in this study, traditional face-to-face interventions, telemedicine, and specialized outpatient services, will benefit more families.

### Study Strengths and Limitations

This study adopted a community-based approach, which is closer to the real world and could be applied to diverse social service settings and target audiences. This study shows that the app is successfully designed for reciprocal interaction between caregivers and pediatric sleep experts to promote caregivers’ behavioral changes to ensure healthy sleep habits among young children. The design of interactive interventions that allow for stepped care, with a specific focus on culturally and family-tailored interventions, and consistent empowerment and support for caregivers resulted in a very high continuity of use while enhancing parenting efficacy. The techniques used in this study may be applicable to other medical or health care domains.

Nonetheless, this study had some limitations. The primary limitation of this study is the moderate sample size. Another limitation is that randomization was not adopted owing to the municipality’s preference for the equality of its citizens. As the design of randomized controlled trials could not be adopted, we cannot exclude the possibility that the improvement in sleep variables in the app use group included the effect of regression to the mean. Therefore, it would be difficult to determine the general effectiveness of the app in this study. We assume that a certain intervention effect was observed after the intervention because significant differences in many sleep variables relative to the video-only group disappeared; however, it is essential to confirm the effect of improvement by randomized controlled trials in the future. Therefore, we should be careful not to overgeneralize the results of this study. Data at the follow-up point were obtained only from the app use group; therefore, the sleep habits of the video-only group at that point are unknown. In addition, because the data in this study were reported by the caregivers, there is a possibility of reporting bias, although the accuracy of the sleep rhythm data entered into the app was confirmed in a previous study by Yoshizaki et al [42]. Furthermore, this community-based trial was conducted in a single community in an urban area. We also note that it is still unclear why changes in sleep parameters predicted accelerated development. Although this study did not include an analysis of the association between the objective parameters of app use such as access history and adherence, the focus on objective parameters should be important for understanding adherence and future development of the app. Further studies should be conducted considering these limitations.

### Conclusions

This study confirmed the long-term continuity of the use of the app and its efficacy in improving sleep habits. In addition, its effects on follow-up maintenance with long-term intervention in the community-based trial was also confirmed. The use of the Nenne Navi app was associated with improved sleep habits and parenting behavior, suggesting an enhanced parenting efficacy in caregivers. The participants’ feedback demonstrated that this effect was supported by the advice that empowered caregivers while encouraging family-tailored, small-step changes in parenting behavior.

The app is expected to be used in sleep medicine and parental education in Japan and is expected to contribute to the expansion of sleep health literacy among families with young children. This app will continue to be implemented in the community as a culturally and individually sensitive, caregiver-supportive sleep education tool. Furthermore, the app could ultimately contribute to improvements in sleep habits and healthy development among Japanese children. Further research must focus on neuroscience to confirm whether this early sleep intervention would lead to more desirable brain development.

## Appendix

Multimedia Appendix 1 Screenshots and the interactive Plan-Do-Check-Act cycle of the app. (A) Snapshot of the app. (B) Screenshots of the top page and data input page. (C) e-Learning content. (D) Interactive Plan-Do-Check-Act cycle of the app.

Multimedia Appendix 2 Demographic information of the study participants.

Multimedia Appendix 3 Developmental age scores for the Kinder Infant Development Scale in each group at the baseline and postintervention stages.

Multimedia Appendix 4 Children’s sleep-wake patterns by group at the baseline, postintervention, and follow-up stages.

## Abbreviations

KIDS: Kinder Infant Development Scale
mHealth: mobile health
