# Supplementary figures and images for: Improving Children’s Sleep Habits Using an Interactive Smartphone App: Community-Based Intervention Study
Source: JMIR Mhealth Uhealth. 2023 Feb 10;11:e40836. doi: 10.2196/40836 (PMC9960041; doi:10.2196/40836)

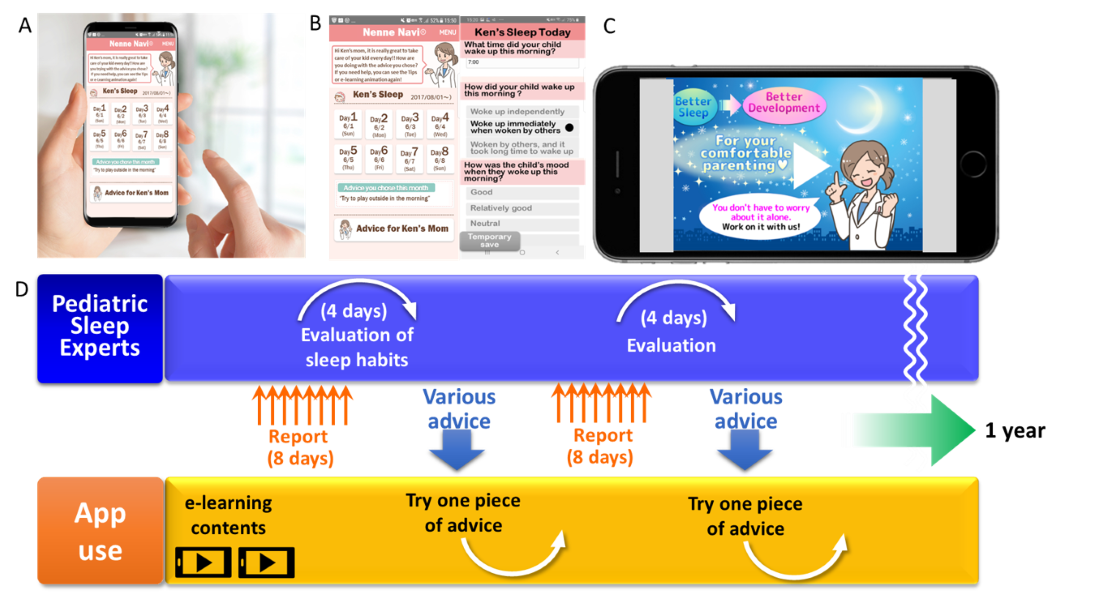

Supplement: Multimedia Appendix 1 [file mhealth_v11i1e40836_app1.png]
