# Supplementary material for: Improving Children’s Sleep Habits Using an Interactive Smartphone App: Community-Based Intervention Study
Source: JMIR Mhealth Uhealth. 2023 Feb 10;11:e40836. doi: 10.2196/40836 (PMC9960041; doi:10.2196/40836)
Supplement: Multimedia Appendix 2 [file mhealth_v11i1e40836_app2.docx]

Multimedia Appendix 2. Demographic information of the study participants

|  | App use | Video only | *P* value |
| --- | --- | --- | --- |
|  | Mean (SD) | Mean (SD) |  |
|  | n=33 | n=34 |  |
| Mother’ age | 34.45 (0.94) | 36.15 (0.94) | .21 |
| Father’ age | 37.65 (1.12) | 38.58 (1.09) | .56 |
| Child age (mos) | 19.61 (0.75) | 19.42 (0.66) | .66 |
| Child gender |  |  |  |
| -Male/Female | 14 / 19 | 13 / 21 | .81 |
| Nursery school / Family care | 4 / 29 | 3 / 31 | .66 |
| Siblings: Yes/No | 16 / 17 | 20 / 14 | .40 |
| Fathers’ education |  |  |  |
| -junior high school | 3 | 1 | .04 |
| -high school | 13 | 7 |  |
| -college/university | 13 | 22 |  |
| -graduate school | 0 | 3 |  |
| -others/n.a. | 4 | 1 |  |
| Mothers’ education |  |  |  |
| -junior high school | 0 | 0 | .76 |
| -high school | 9 | 8 |  |
| -college/university | 21 | 22 |  |
| -graduate school | 0 | 2 |  |
| -others/n.a. | 3 | 2 |  |
| Family income (Yen) |  |  |  |
| < 3,000,000 | 1 | 0 | .67 |
| 3,000,000 ~ 5,000,000 | 9 | 5 |  |
| 5,000,000 ~ 7,000,000 | 10 | 14 |  |
| 7,000,000 ~ 10,000,000 | 4 | 6 |  |
| 10,000,000 < | 2 | 1 |  |
| Not available | 7 | 8 |  |
