# Supplementary material for: Improving Children’s Sleep Habits Using an Interactive Smartphone App: Community-Based Intervention Study
Source: JMIR Mhealth Uhealth. 2023 Feb 10;11:e40836. doi: 10.2196/40836 (PMC9960041; doi:10.2196/40836)
Supplement: Multimedia Appendix 3 [file mhealth_v11i1e40836_app3.docx]

Multimedia Appendix 3. Developmental age scores for the Kinder Infant Development Scale in each group at the baseline and postintervention stages.

|  | App use | | Video only | |
| --- | --- | --- | --- | --- |
|  | Baseline | Postintervention | Baseline | Postintervention |
|  | Mean (SD) | Mean (SD) | Mean (SD) | Mean (SD) |
| Physical motor | 19.70 (1.64) | 32.70 (2.72) | 20.27 (1.94) | 32.30 (4.35) |
| Manipulation | 21.58 (1.81) | 31.39 (2.74) | 21.15 (1.99) | 33.03 (2.30) |
| Receptive language | 23.48 (3.70) | 36.85 (1.84) | 22.97 (4.22) | 35.76 (3.74) |
| Expressive language | 21.76 (2.17) | 31.18 (3.15) | 20.82 (3.31) | 31.70 (3.59) |
| Language concepts | 16.76 (1.50) | 30.24 (4.63) | 16.79 (3.33) | 29.73 (5.27) |
| Social relations with children | 20.03 (2.60) | 30.76 (4.78) | 19.88 (2.87) | 32.15 (4.08) |
| Social relations with adults | 24.39 (3.62) | 34.39 (3.01) | 23.70 (4.54) | 33.82 (3.21) |
| Discipline | 24.15 (3.11) | 30.33 (2.54) | 23.15 (3.00) | 30.00 (3.01) |
| Feeding | 23.52 (3.01) | 31.36 (3.42) | 23.21 (4.07) | 31.61 (3.46) |
