# Supplementary material for: Improving Children’s Sleep Habits Using an Interactive Smartphone App: Community-Based Intervention Study
Source: JMIR Mhealth Uhealth. 2023 Feb 10;11:e40836. doi: 10.2196/40836 (PMC9960041; doi:10.2196/40836)
Supplement: Multimedia Appendix 4 [file mhealth_v11i1e40836_app4.docx]

Multimedia Appendix 4. Children’s sleep-wake patterns by group at the baseline, postintervention, and follow-up stages.


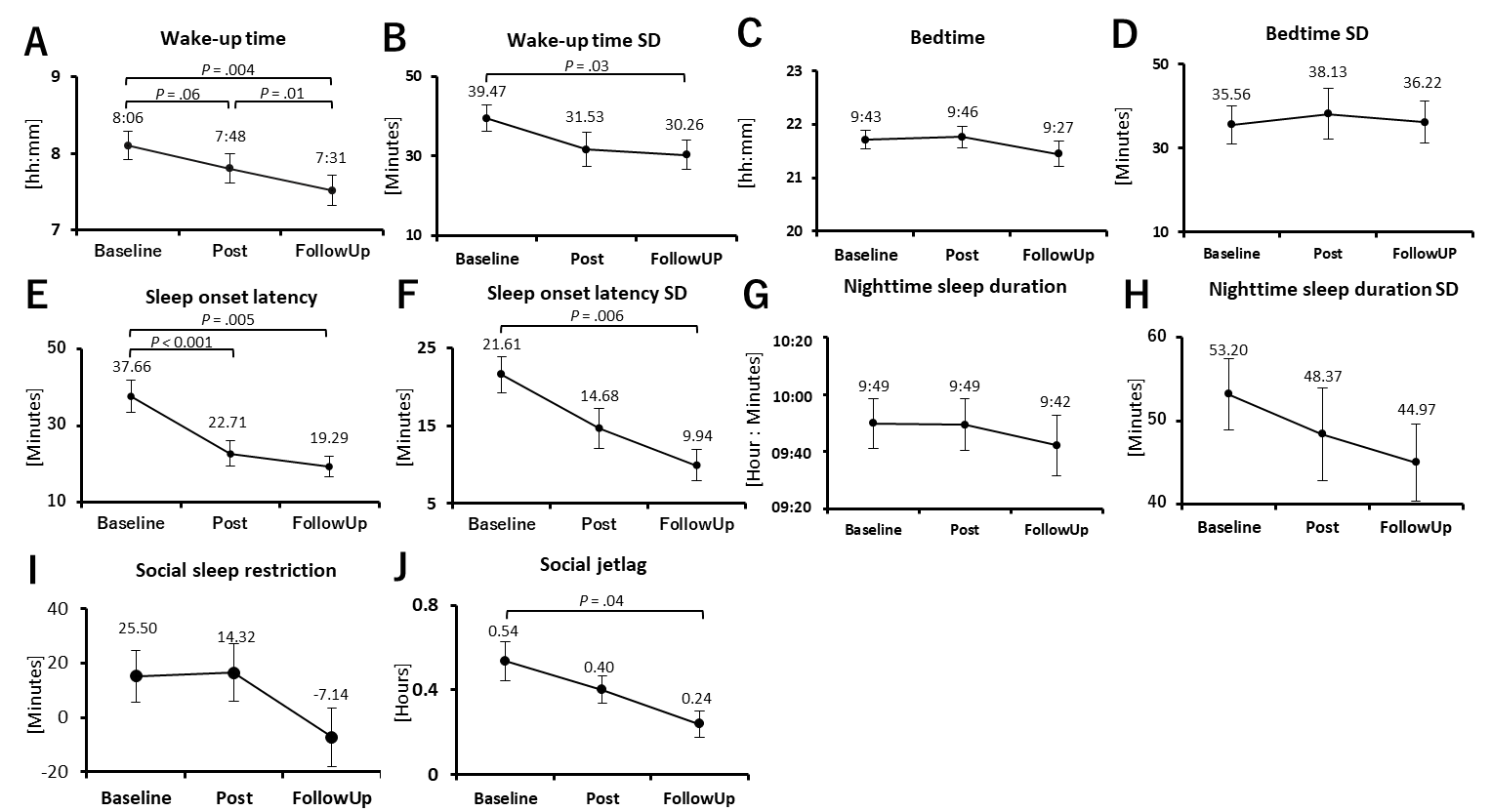


The evaluation of the impact of the intervention involved a single-group repeated measures design with three intervention assessment stages: 1) baseline, (2) 13th month (postintervention), and (3) 6 months after postintervention (follow-up). A single-factor repeated measures analysis of variance (ANOVA) was used to identify the change on each sleep habit variable of the app usage group between these timepoints.

Firstly, the result showed a significant advance in wake-up time (A; F(1,27)=9.703, *P*=.004). Multiple comparisons with the Bonferroni method showed a significant trend from baseline to postintervention (baseline>postintervention, *P*=.06) , and a significant difference from baseline to follow-up (baseline>follow-up, *P*=.004) and from postintervention to follow-up (postinterention > follow-up, *P*=.01).

Next, a significant reduction in wake-up time SD was confirmed (B; F(1,27)=8.072, *P*=.008). Multiple comparisons with the Bonferroni method showed a significant reduction from baseline to follow-up (baseline>follow-up, *P*=.03) .

Third, a significant reduction in sleep onset latency was also confirmed (E; F(1,27)=12.244, *P*=.002). Multiple comparisons with the Bonferroni method showed a significant reduction from baseline to postintervention (baseline>postintervention, *P*<.001), and baseline to follow-up (baseline > follow-up, *P*=.005) .

Fourth, a significant reduction in sleep onset latency SD was confirmed (F; F(1,27)=11.745, *P*=.002). Multiple comparisons with the Bonferroni method showed a significant reduction from baseline to follow-up (baseline>follow-up, *P*=.006) .

Further, a significant reduction in social jetlag was confirmed (J; F(1,27)=7.048, *P*=.01). Multiple comparisons with the Bonferroni method showed a significant reduction from baseline to follow-up (baseline>follow-up, *P*=.040) .

Finally, there were no significant difference for bedtime (C; F(1,27)=1.232, *P*=.28), bedtime SD (d; F(1,27)=0.010, *P*=.92), nighttime sleep duration (G; F(1,27)=0.554, *P*=.46), nighttime sleep duration SD (H; F(1,27)=1.581, *P*=.22), and social sleep restriction (I; F(1,27)=2.122, *P*=.16).
